# Associations between household garden size and childhood obesity in Wales, UK.

**DOI:** 10.23889/ijpds.v7i3.1983

**Published:** 2022-08-25

**Authors:** Rhodri Johnson, Amy Mizen, Rowena Bailey, Lucy Griffiths, Richard Fry

**Affiliations:** 1 Swansea University

## Objectives

Physical inactivity directly contributes to the global issue of obesity. Small-scale garden-based interventions have positively impacted on children’s physical activity levels. However, no studies have objectively measured household gardens and assessed associations with childhood obesity on a population scale. We linked garden measures with administrative data for a national population.

## Approach

Our cross-sectional study examined the relationship between garden size and BMI in 154,444 children aged 5 years living in Wales between 2013-2019. We linked garden size at the home location with individual-level BMI measurements and socio-demographic data in the SAIL databank; a secure research environment. BMI measures were derived from the Child Measurement Programme and were standardised using the LMS method. We calculated descriptive statistics for our cohort and used Generalised Additive Models to investigate associations between garden size and BMI. Our results include adjustment for confounding variables such as deprivation. We stratified our analysis by gender and rurality.

## Results

Our cohort consisted of 52% male and 49% females. 18% lived in the most affluent quintile and 28% lived in the most deprived quintile. 74% of the cohort lived in urban areas whilst 26% lived in rural areas. Average garden size was 176m2 and the mean number of individuals living in the home was 4. We found a non-linear statistically significant negative association between garden size and BMI (edf = 2.37, p < 0.01). When we stratified our analysis by gender, for males we found a linear statistically significant negative association between garden size and BMI (edf = 1, p < 0.01). For females we found a non-linear statistically significant association between garden size and BMI (edf = 2.67, p < 0.05).

## Conclusion

Our study is the first to link routinely measured objective household variables with BMI data for a national cohort. Our results suggest that garden-based interventions could be focussed on to increase physical activity in children. Further research is required to investigate the pathways between garden, physical activity and reducing obesity.

